# Designing 3D Anode Based on Pore‐Size‐Dependent Li Deposition Behavior for Reversible Li‐Free All‐Solid‐State Batteries

**DOI:** 10.1002/advs.202203130

**Published:** 2022-08-10

**Authors:** Se Hwan Park, Dayoung Jun, Gyu Hyeon Lee, Seong Gyu Lee, Ji Eun Jung, Ki Yoon Bae, Samick Son, Yun Jung Lee

**Affiliations:** ^1^ Department of Energy Engineering Hanyang University Seoul 04763 Republic of Korea; ^2^ Advanced Battery Development Group Hyundai Motor Company Hwaseong‐si Gyeongi‐do 16082 Republic of Korea

**Keywords:** all‐solid‐state‐battery, anode, anodeless, diffusional coble creep, lithium metal, lithium‐free, morphology

## Abstract

Li‐free all‐solid‐state batteries can achieve high energy density and safety. However, separation of the current collector/solid electrolyte interface during Li deposition increases interfacial resistance, which deteriorates safety and reversibility. In this study, a reversible 3D porous anode is designed based on Li deposition behavior that depends on the pore size of the anode. More Li deposits are accommodated within the smaller pores of the Li hosting anode composed of Ni particles with a granular piling structure; this implies the Li movement into the anode is achieved via diffusional Coble creep. Surface modification of Ni with a carbon coating layer and Ag nanoparticles further increases the Li hosting capacity and enables Li deposition without anode/solid electrolyte interface separation. A Li‐free all‐solid‐state full cell with a LiNi_0.8_Mn_0.1_Co_0.1_O_2_ cathode shows an areal capacity of 2 mAh cm^−2^ for retaining a Coulombic efficiency of 99.46% for 100 cycles at 30 °C.

## Graphical abstract

This work clarifies that the mechanism of Li movement into the porous anode in all‐solid‐state batteries is via diffusional Coble creep and facilitated in smaller pores. From this understanding, a highly reversible 3D porous anode with proper size and surface property is fabricated. The anode accommodates Li deposits within porous architecture without anode/solid‐electrolyte interface separation and enables stable cycling.

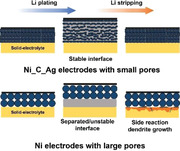

## 1 Introduction

Lit metal‐based all‐solid‐state batteries (ASSBs) can potentially combine the high energy of Li metal anodes and the safety of ASSBs.^[^ ^1^, ^2^ ^]^ Among Li metal‐based ASSBs, Li‐free or anodeless ASSBs are considered optimal battery configurations because of their high energy density and economic advantages attributed to the absence of Li metal during the battery assembly process.^[^ ^3^, ^4^, ^5^, ^6^ ^]^ Despite these merits, the short cycling life and low Coulombic efficiency (CE) hinder the commercial application of anodeless ASSBs.^[^ ^7^, ^8^ ^]^ One reason for this inferior performance is the unstable interface. The separation of the anode/SE interface during Li deposition degrades the interfacial contact between the anode and SE; therefore, the interfacial resistance increases as Li deposition and stripping are repeated. The increased interfacial resistance can lead to inhomogeneous Li deposition, dendritic Li growth, and internal short circuits. Owing to this high interfacial barrier, operation of ASSBs at room temperature is a formidable task until now. Thus, maintaining the anode/SE interface without separation is crucial for ASSBs to operate with high reversibility at room temperature.

A 3D porous anode with appropriate architecture and materials can accommodate Li deposits within its free volume and maintain intimate interfacial contact with the SE during repeated Li deposition and stripping. Furthermore, such 3D anodes can mitigate dendritic Li growth and suppress internal short circuits.^[^ ^6^, ^9^, ^10^ ^]^ However, the intrinsic immobile property of SE makes designing suitable 3D porous anodes more complicated than the anode in the cell using a liquid electrolyte. While the liquid electrolyte can infiltrate into the porous structure and deliver Li ions into the free volume of the 3D architecture, the SE can neither completely fill the free volumes of 3D anode, which should be empty to accommodate the Li deposits, nor freely move into the structure.^[^ ^3^ ^]^ Therefore, filling the 3D porous anodes with Li deposits is very challenging in ASSBs. In fact, 3D hosts for ASSBS are only a few, and their success at room temperature is particularly rare.

A porous framework composed of SE can transfer Li ions by itself (through the frame) into the pores of the anode; the oxide SEs were used to construct a 3D architecture.^[^ ^10^, ^11^, ^12^, ^13^ ^]^ Li ions were reduced on the surface of SE and metallic Li was electrochemically deposited within the 3D framework. The 3D porous SE framework anode cycled stably, however, the cells showed unstable voltage profiles in the Li‐free configuration (i.e., the cell with zero‐excess Li metal). When carbon tubules with a width of 100 nm were used to host Li metal, Li deposits advanced and retracted inside the tubules during Li plating and stripping via plastic deformation, specifically diffusional Coble creep.^[^ ^9^, ^14^ ^]^ The anode of hollow carbon tubules with a ZnO‐coated inner surface was highly reversible in repeated Li plating and stripping in an anodeless configuration. However, the complicated anode fabrication processes, which include chemical vapor deposition (CVD) and atomic layer deposition (ALD), involve high manufacturing costs and hinder mass production for commercial applications.

Various approaches, including 3D porous anodes, have been introduced to control the Li deposition behavior and improve the reversibility of anodeless ASSBs; however, the mechanism and conditions for determining the Li deposition behavior remain unclear. Particularly, Li metal moved inside the porous carbon tubules like working fluid via mechanical deformation called Coble creep.^[^ ^9^, ^17^ ^]^ The creep deformation occurs below the yield strength and is sensitive to temperature. Due to its low melting temperature, significant creep can occur for Li at battery‐relevant conditions. Creep is categorized into two types; diffusional and displacive (dislocation). Diffusional creep occurs as atoms diffuse through grain boundary (or interfaces) or bulk crystals at low stresses, and depending on the pathway, each creep mechanism is called Coble and Nabarro‐Herring creep, respectively. Besides, displacive creep takes place by the movement of dislocations at high stresses. Diverse factors such as temperatures, pressures, stress, and feature sizes affect strain rate of each creep mechanism and determine the dominant mechanism.^[^ ^14^, ^15^, ^16^ ^]^ In the literature, it has been suggested that the inner diameter of the pores should be less than a few hundred nanometers (≈200 nm) to enable Li deposition based on the diffusional Coble creep mechanism; further, the main mechanism of deformation changes for larger pores.^[^ ^9^ ^]^ This critical pore size for Coble creep to occur was estimated simply based on the tensile deformation experiment of the Sn nanowire;^[^ ^17^ ^]^ however, the actual critical dimension for Li deposition into the 3D porous anode is yet to be experimentally proven. The Li deposition behavior that depends on the architecture and materials of the 3D anode should be clarified to design a suitable 3D anode for highly reversible and long‐cycling ASSBs operating at room temperature.

We closely investigate the Li deposition behavior that depends on the pore size of the anode using a 3D porous anode fabricated by piling up Ni particles of three different diameters. As the pore size decreased (i.e., as the particle size decreased), a larger amount of Li deposits was accommodated within the anode among the total 2 mAh cm^−2^ deposition. The Li not accommodated in the porous anode separated SE and 3D porous host. We expected that Li deposits filled the pores via plastic deformation because Ni particles do not have Li‐ion conducting properties, and the size‐dependent Li deposition behavior confirmed that the main mechanism of Li accommodation is through diffusional Coble creep. The surface of the Ni particles was further modified with a carbon‐coating layer and Ag nanoparticles. This modification allowed the 3D anode host to accommodate larger amounts of Li deposits of 2 mAh cm^−2^ (the total amount), presumably because of the high Li mobility endowed by C and particularly Ag, as well as the lower energy barrier for Li nucleation enabled by Ag. Because the entire Li deposits advanced into the 3D framework, the SE and 3D porous host were not separated and maintained intimate contact between these two components. Thus, modified 3D anode had lower interfacial resistance than that of the anode of the planar Ni foil and pristine Ni particles. This 3D anode presented remarkably improved reversibility in Li deposition/stripping. Furthermore, the 3D anode developed in this study enabled the stable operation of full‐cell ASSBs in anode‐free configuration, notably at room temperature. The Li‐free all‐solid‐state full‐cell equipped with our 3D anode coupled with a LiNi_0.8_Mn_0.1_Co_0.1_O_2_ (NCM 811) cathode demonstrated operation at room temperature (30 °C) with an initial discharge capacity of 2 mAh cm^−2^ and an average CE of 99.47% for 100 cycles.

## 2 Results and Discussion

### 2.1 Fabrication of 3D Porous Electrode with Different Empty Space Diameters

**Figure 1:**
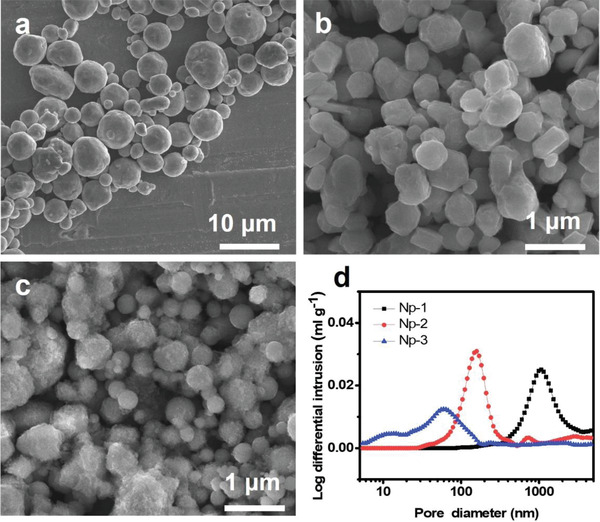
SEM images of Ni particles with different diameters: a) Np‐1, b) Np‐2, and c) Np‐3. d) Pore size distribution of Ni particle electrode obtained by mercury porosimetry.

3D porous electrodes prepared by piling Ni particles of three different sizes were used to investigate the Li deposition behavior depending on the pore size of the porous anode. **Figure** **1** shows the scanning electron microscopy (SEM) images of the Ni particles. All Ni particles have spherical morphology, and the corresponding average diameters are 3.58 µm (Np‐1), 504 nm (Np‐2), and 314 nm (Np‐3) (Figure S1, Supporting Information). 3D porous anodes were fabricated by casting Ni particles on the Ni foil current collector (CC), and the thickness of the resulting Ni particle layer was 20 µm for Np‐1 and 15 µm for both Np‐2 and Np‐3 (Figure S2a–c, Supporting Information). Both cross‐sectional (Figure S2a–c, Supporting Information) and top‐view (Figure S2d–f, Supporting Information) SEM images of the electrodes confirmed the porous structure with numerous empty pores between Ni particles; all Ni particles maintained their spherical morphology (Figure S2d–f, Supporting Information). The pore size distributions of each electrode were investigated using mercury porosimetry. As shown in Figure 1d, the pore diameter of the electrodes increased with particle size. The measured average pore diameters were 1.11 µm, 163 nm, and 56.8 nm for Np‐1, Np‐2, and Np‐3, respectively.

### 2.2 Li Deposition Behavior Based on Pore Size of the Anode

**Figure 2:**
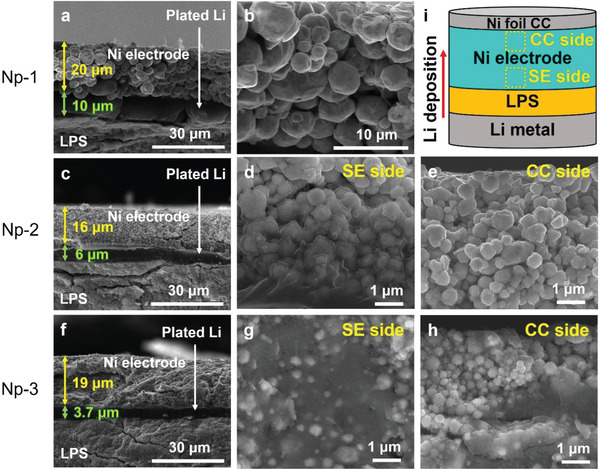
a–h) SEM images of Li deposited 3D Ni electrodes plated to the capacity of 2 mAh cm^−2^. (a,b) for Np‐1, (c–e) for Np‐2, and (f–h) for Np‐3. (d,g) and (e,h) represent the Ni electrode of solid electrolyte and CC side, respectively. i) Schematic illustration of ASSB employed in the experiment. The dashed rectangles are the position of SE and CC sides, respectively.

Li was electrochemically plated on the Ni‐based electrode in half‐cell ASSBs to investigate the Li deposition behavior depending on the pore size of the porous anode, as depicted in **Figure** **2**i. Ni particle‐based 3D porous anodes composed of Np‐1, 2, and 3, and Li metal foil were used as the working and counter electrodes, respectively. Lithium thiophosphate (Li_3_PS_4_, LPS) was employed as the SE; the cells were operated under a uniaxial pressure of 5 MPa at a temperature of 45 °C. Li metal was plated at a current density of 0.5 mA cm^−2^ to a capacity of 2 mAh cm^−2^. Figure 2a–h shows cross‐sectional SEM images of the cells after Li deposition. The Li deposit layers between the Ni‐based anode and SE were observed for all three cells; the presence of a Li layer at the anode/LPS interface was confirmed by energy‐dispersive X‐ray spectroscopy (EDS) elemental mapping (Figure S3, Supporting Information). While the Li element in the Li deposit layer cannot be identified by EDS, the positions of Ni‐based anode and SE are clearly marked by the elemental mapping of Ni and S. The dark space between the Ni‐based anode and LPS SE must be the deposited Li layer. Figure 2 shows the Li deposition behavior, which includes the thickness of the deposited Li layer at the anode/LPS interface and distribution of Li deposits within the pores of the anodes, and it showed clear differences depending on the pore size of the anodes. The thickness of the Li layer at the anode/LPS interface increased with increasing pore size, which implies that the porous anode of the smaller pore hosted a larger amount of Li because the total capacity deposited was the same at 2 mAh cm^−2^ for all three cells. The thicknesses of the Li layer at the anode/SE interface were 10, 6, and 3.7 µm for the cell employing Np‐1, 2, and 3 electrodes, respectively. Considering the theoretical thickness of 2 mAh cm^−2^ Li deposits is approximately 10 µm and the Li layer thickness at the anode/SE interface of the cell with the Np‐1 anode was 10 µm, it is believed that the entire Li deposit was plated at the anode/LPS interface rather than within the empty space of the Np‐1 anode. Further, as shown in Figure 2b, Li deposits were not found within the pores of the Np‐1 anode. Ni particles in the Np‐1 anode maintained their neat morphology after Li plating compared to the pristine state; the thickness of the electrode remained unchanged. Each Ni particle maintained its smooth surface, and the voids of the anode remained empty. Thus, all Li deposits exist at the anode/LPS interface. In contrast, Li deposits were observed not only at the anode/LPS interface but also within the empty space of the porous anode for cells with smaller pore diameters (cells employing the anode of Np‐2 and 3). In the cell with the Np‐2 anode, the thickness of the Li layer at anode/SE decreased to 6 µm, which is smaller than 10 µm (the theoretical thickness of 2 mAh cm^−2^ Li deposits) (Figure 2c). Furthermore, the Li metal partially filled the empty space of the anode wrapping the Ni particles on the SE side of the anode (Figure 2d). However, at the CC side of the anode (far from the SE), Li deposits were not observed, and Ni particles maintained their initial morphology without changing from the pristine state (Figure 2e). This suggests that Li advanced only to the SE part of the porous anode. In the cell with the Np‐3 anode, the thickness of the Li layer at the interface of anode/SE decreased further to 3.7 µm (Figure 2f), which implies that a larger amount of Li deposits exists within the 3D porous anode. Interestingly, as shown in Figure 2g,h, Li metal filled the empty space of the anode at both the SE and CC sides. The Li metal wrapped Ni particles, and in some parts, Ni particles appeared to be embedded completely in the Li matrix. In a similar context, when the top surface of the 3D porous Ni electrode was examined after delaminating the Ni foil CC, as described in Figure S4h, Supporting Information, Li deposits advanced to and filled the void of the top surface of the porous electrode in the cell with the Np‐3 anode (Figure S4e,f, Supporting Information), whereas no Li penetrated to the top of the porous electrode. Moreover, the anode surface maintained its pristine state of porous morphology in the cells with the Np‐1 (Figure S4a,b, Supporting Information) and Np‐2 anodes (Figure S4c,d, Supporting Information). This result indicated that the entire Np‐3 anode participated in accommodating the Li deposits. In Figure 2f, it is worthy of special notice that the thickness of the Np‐3 anode increased from 15 to 19 µm after Li deposition, which implies Li deposits expanded the distance between Ni particles. During the Li deposition into the Np‐3 anode, Li deposits filled the pores of anode first and subsequently, expanded the anode before being plated at the interface of anode/SE. Li deposits filled the entire pores of the anode after 0.5 mAh cm^−2^ Li plating (Figure S5, Supporting Information) and expanded the Np‐3 anode to the thickness of 18 µm after 1 mAh cm^−2^ deposition, without Li plating at SE surface (Figure S6, Supporting Information). The thickness of the anode did not change for Np‐1 because no Li resided within the 3D Ni anode (20 µm before and after Li plating) and that of Np‐2, which partially accommodated Li, increased by 1 µm after Li deposition (15 to 16 µm before and after Li plating, respectively).

Given that Ni particles do not form an alloy phase with Li and have infinitesimal Li ion conductivity, it is highly likely that the electrochemical reduction reaction of Li ions occurs at the anode/LPS interface, where Li ions and electrons are transported through LPS and Ni particles, respectively. Subsequently, metallic Li grows at the interface of the 3D anode/SE and generates mechanical stress because of volume expansion accompanied. Electrochemically generated stress can either separate the 3D anode/SE interface by depositing the Li layer, or the stress can be relieved by moving Li deposits into the empty space of Ni‐based anodes. As mentioned in the introduction, Li metal reversibly grew out of and retracted within the hollow carbon tubules of 100 nm width and 10–100 µm length during the Li plating and stripping, respectively, in the previous literature.^[^ ^14^ ^]^ The diffusional Coble creep was identified as the dominant mechanism of Li movement in this system using in situ transmission electron microscopy (TEM). Further, Coble‐creep‐based Li deposition/stripping was considered feasible for other 3D open porous architectures composed of electrochemically stable materials against Li, which includes Ni. Therefore, we surmised that Li deposits flowed to the empty space of the Ni‐based 3D porous anode via diffusional Coble creep in this study. Further, the Li deposition behavior showed the penetration of Li into the 3D porous Ni anode depending on the pore size of the anode. A previous study on the transition of the deformation mechanism in Sn nanorods depending on rod size is in line with our assumption.^[^ ^17^ ^]^ When the diameter of the Sn rod was reduced from 450 to 130 nm, the deformation mechanism under uniaxial compression changed from displacive plasticity to Coble diffusion, and plastic flow occurred at considerably lower stress values in the sample with a smaller diameter. Sn and Li metals are expected to show similar deformation behavior at room temperature because Sn has a similar homologous temperature (T/T_m_, T_m_ is the melting temperature and T at room temperature) as Li at approximately 0.6,^[^ ^9^ ^]^ and the transition of the deformation mechanism upon decreasing feature size of Li (here, the pore size) can occur. When the pore size is large (average 1.11 µm for Np‐1), Li deposits presumably deform via the mechanism of displacive plasticity, which requires higher stress than the working pressure of the cell and normal hydrostatic pressure generated by Li deposition.^[^ ^9^ ^]^ Thus, Li deposits cannot grow into the empty space of the anode, and the initial stationary growth will build up stress because of volume expansion. Eventually, this accumulated stress separates the SE and the porous anode. Li yielded and horizontal plastic deformation occurred by depositing Li at the anode/SE interface.^[^ ^5^ ^]^ On the other hand, within the anode with a smaller pore size (average 163 and 56.8 nm for Np‐2 and Np‐3, respectively), Li movement into the porous 3D Ni anode could occur via diffusional Coble creep, which requires less stress. Therefore, Li deposits could fill (at least partially) the empty space of the anode under a smaller stress.

### 2.3 Fabrication of Ni_C_Ag to Improve the Mobility of Li within 3D Porous Anode

**Figure 3:**
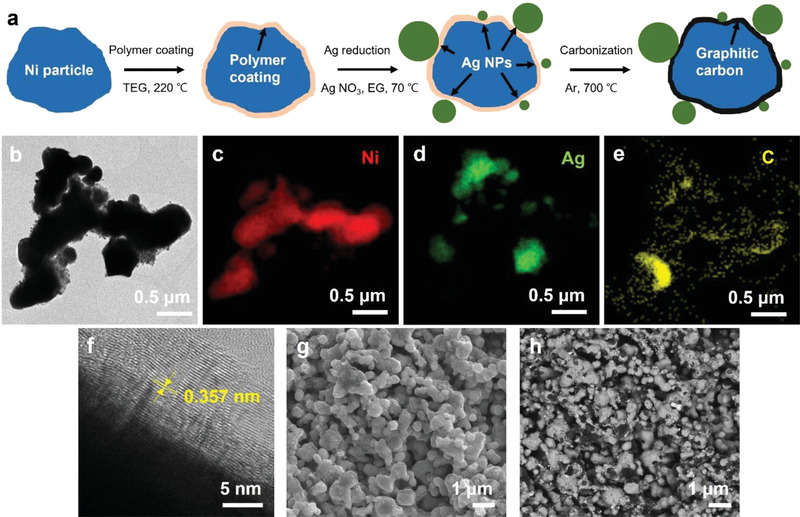
a) Schematic illustration of Ni_C_Ag synthesis process. b) TEM image of Ni_C_Ag powder and corresponding EDS elemental mappings for c) Ni, d) Ag, and e) C. f) High‐resolution TEM image of carbon coating layer of Ni_C_Ag powder. SEM image of Ni_C_Ag powder for g) secondary electron mode and h) BSE mode.

Although the largest amount of Li deposits was accommodated within the porous structure of Np‐3 among three Ni‐based anodes, a part of the Li metal layer remained at the interface of the anode/LPS SE. Li deposited at the interface between the anode and LPS eventually results in interfacial delamination and loss of interfacial adhesion during repeated Li plating and stripping. The entire Li deposit should be accommodated within the 3D anode to maintain interfacial contact during operation. For this purpose, we coated Np‐3 particles with a carbon layer and decorated them with Ag nanoparticles on the surface of the Np‐3 particles. As illustrated in **Figure** **3**a, the polymer coating was formed first on the surface of Np‐3 particles, and silver nitrate was reduced by ethylene glycol under basic conditions. Therefore, Ag nanoparticles were precipitated on polymer‐coated Ni particles. The final annealing in an Ar atmosphere formed a graphitic carbon coating on the surface of the Ni particles. The mass ratio of Ag in Ni_C_Ag was estimated as 8.1% based on the reported yield of Ag nanoparticles synthesis from the reduction of silver nitrate in basic conditions.^[^ ^18^ ^]^

Figure 3b–e shows the TEM image and corresponding EDS elemental mappings of carbon‐coated Np‐3 decorated with Ag nanoparticles (Ni_C_Ag). The EDS mapping confirmed that the carbon layer uniformly coated the Np‐3 particles, and the Ag nanoparticles were well dispersed around Np‐3. A graphitic carbon layer with 0.357 nm interplanar spacing was found on the surface of Np‐3 particles in the high‐resolution TEM image (Figure 3f); this d‐spacing of the carbon layer was consistent with previous reports for graphene.^[^ ^19^, ^20^ ^]^ Although Ag and Ni particles were not distinguishable in the SEM image of the secondary electron mode (Figure 3g), the Ag particles appeared brighter compared to the Ni in the backscattered electron (BSE) images (Figure 3h). Bright and dark particles were homogeneously dispersed in the BSE image, which confirms that Ag particles were uniformly attached to the surface of the Ni particles. Furthermore, the EDS maps of Ni_C_Ag verified that each element was well dispersed (Figure S7, Supporting Information).

### 2.4 Li Deposition Behavior within the Ni_C_Ag Anode

**Figure 4:**
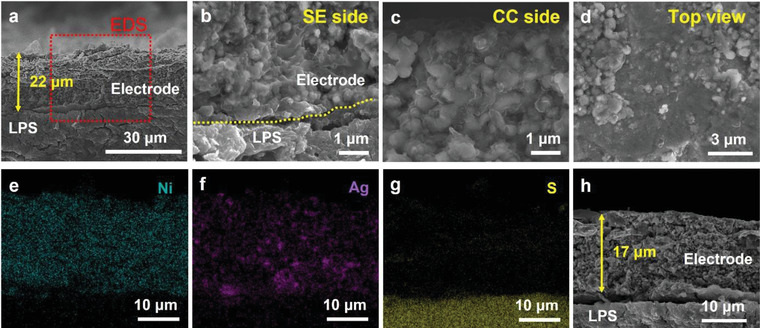
a–c) Cross section and d) surface SEM images and e–g) EDS elemental mappings of Li deposited Ni_C_Ag electrode plated to the capacity of 2 mAh cm^−2^: (b) For solid electrolyte side, (c) for CC side, (d) for surface of CC side observed after delaminating Ni foil CC (refer to the schematic illustration in Figure S4h, Supporting Information). EDS elemental mappings for (e) Ni, (f) Ag, and (g) S. h) Cross‐sectional SEM image of Ni_C_Ag electrode after 2 mAh cm^−2^ Li plating and stripping to 1 V.

The Li metal was plated onto Ni_C_Ag under the same conditions as the previous experiment by employing Np‐1, 2, and 3 anodes (current density of 0.5 mA cm^−2^, operating temperature of 45 °C, and uniaxial pressure of 5 MPa to a capacity of 2 mAh cm^−2^) to compare the Li deposition behavior within the Ni_C_Ag anode with that of the pristine Ni‐based anode. In contrast to the untreated Ni particle‐based anode, Li was not deposited at the interface of anode/LPS (**Figure** **4**a) and the Ni_C_Ag anode maintained intimate contact with LPS (Figure 4b, high magnification image of anode/LPS interface) after Li plating. The Li deposits densely filled the empty spaces of the anode on both the SE and CC sides (Figure 4b,c). In the top‐view SEM image of the CC side of the anode, after peeling off the CC (Figure 4d), individual Ni_C_Ag anode particles were embedded in the Li deposits. The corresponding EDS elemental mappings of the cross‐sectional SEM image indicated that the Li layer was not formed at the interface of the anode/LPS and Ni_C_Ag 3D porous anode, and the LPS SE were not separated (Figure 4e–g). Further, Ag was homogeneously dispersed throughout the entire anode (Figure 4f). Based on the SEM and EDS results, we concluded that all Li deposits were stored within the Ni_C_Ag anode; the entire Ni_C_Ag anode participated in accommodating the Li deposits. The total thickness of the Ni_C_Ag anode increased after Li deposition from 14 µm (Figure S8b, Supporting Information) to 22 µm (Figure 4a) as the Li metal filled the empty space of the anode and expanded the particle–particle distance. When Ni_C_Ag anode was observed after 0.5 (Figure S9, Supporting Information) and 1 mAh cm^−2^ (Figure S10, Supporting Information) of Li deposition, it was confirmed that the Li deposits filled the entire Ni_C_Ag anode first, and increased the thickness of anode. As a larger amount of Li deposits was accommodated within the Ni_C_Ag, the thickness of the anode was increased further by 8 µm compared to that of Np‐2 and 3, wherein the porous anode thickness was increased by 1 and 4 µm, respectively. After Li stripping to 1 V, the thickness of the anode decreased to 17 µm (Figure 4h). As the Li metal left the anode, the Ni_C_Ag anode considerably recovered its original state, but the thickness has increased by 3 µm compared to its pristine state. Although some deposited Li remained in the Ni_C_Ag anode, the 2 mAh cm^−2^ Li could grow into and be stripped out from the 3D porous Ni_C_Ag anode while maintaining the intimate contact between the anode and the LPS.

The improved Li storage capacity of the Ni_C_Ag anode can be explained as follows: 1) C and especially Ag electrochemically react with Li‐ion above 0 V (which forms lithiated carbon and Li‐Ag alloy) before metallic Li formation and thus Li‐ion can be transported to the 3D Ni_C_Ag porous anode before the Li deposition at the SE/anode interface at < 0 V (because of the overpotential).^[^ ^14^, ^21^ ^]^ In addition, the high Li ion diffusion coefficient of lithiated carbon and Li‐Ag alloy could facilitate further Li ion reduction within the pores of 3D anode, and thus, Li deposition can occur within the porous 3D Ni anode.^[^ ^22^ ^]^ Indeed, the Li‐ion diffusion coefficient of lithiated C and Li‐Ag alloy is considerably higher than those of the bulk Li metal (1 × 10^−8^ cm^2^ s^−1^ for LiC_6_ and Li‐Ag and 5.6 × 10^−11^ cm^2^ s^−1^ for Li).^[^ ^14^, ^23^ ^]^ SE is the exclusive Li ion pathway within the cell with a bare Ni anode; however, the lithiated C and Li‐Ag alloy in Ni_C_Ag anode could transfer Li‐ion within the porous anode and facilitate the electrochemical reduction of Li‐ion within the anode, which suppresses the separation of the anode/SE interface. A previous study demonstrating different Li deposition behavior on Ni‐and Au‐coated membrane electrodes is in line with our explanation.^[^ ^24^ ^]^ Li deposits extruded from the SE surface in the Ni‐coated membrane electrode because of the lack of the Li diffusion through Ni, whereas Li was deposited on the inner wall of the Au‐coated micropores of the membrane filter, which implies Li diffusion in the Au layer.

2) Lithiophilic C and Ag could facilitate the movement of Li via diffusional Coble creep. The anode material can be an important factor for diffusional Coble creep because the mobility of Li ions in the anode is significantly influenced by the anode surface. Compared to Ni, lithiated C has a higher Li ion conductivity and better lithiophilicity, which could lead to better interfacial Li movement. More importantly, employing lithiophilic Ag may remarkably improve the Li adatom mobility‐boosting diffusional Coble creep. For the Ni anode, the Li layer formed on the Ni particles contains vacancies and disorders because of the lattice mismatch between Li and Ni.^[^ ^25^ ^]^ These defects increase the energy barrier for surface diffusion, which induces sluggish Li adatom mobility on the metal surface. In the binary phase diagram, Ag not only reacts with Li‐generating Li_x_Ag alloy phases, but also has a solubility in Li (≈9 at.%@145.5 °C). The solid solution phase with dissolved Ag (denoted as Li (Ag)) has a structure identical to that of pure metallic Li, and it has been reported to serve as a buffer for the subsequent Li deposition.^[^ ^21^ ^]^ The Li adatom mobility on this Li (Ag) phase would be as high as that on the pure Li metal surface.

3) The solubility of Ag in Li allows the formation of a solid solution phase Li (Ag) before the formation of a pure Li phase, and the Li (Ag) surface layer can serve as a buffer layer for Li deposition, which lowers the nucleation energy barrier for Li.^[^ ^21^ ^]^ The Ag in our study could thus lower the nucleation energy barrier for Li in the 3D anode. The facilitated Li movement (Li‐ion diffusion and Li adatom mobility) and reduced energy barrier for Li nucleation synergistically combined in the Ni_C_Ag anode showed improved Li storage capacity in the 3D Ni_C_Ag host.

### 2.5 Electrochemical Performance of Ni_C_Ag

**Figure 5:**
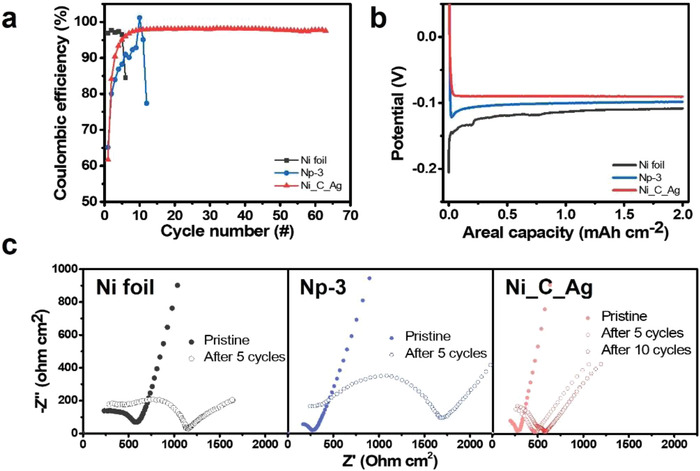
a) Variation of coulombic efficiency during the repeated Li plating and stripping on Ni foil, Np‐3, and Ni_C_Ag electrode. b) 1st Li deposition voltage profiles of Ni foil, Np‐3, and Ni_C_Ag electrode. c) EIS analysis for Ni foil, Np‐3, and Ni_C_Ag electrode at pristine state before test, and after repeated Li plating and stripping.

We first examined the electrochemical performance of Ni‐based electrodes in a half‐cell configuration to demonstrate the effect of the 3D porous architecture and carbon coating with Ag decoration. Li metal was plated on the anodes of 2D Ni foil, a 3D porous anode of Np‐3, and Ni_C_Ag with a curtailing capacity of 2 mAh cm^−2^ and stripped to 1 V at a current density of 0.5 mA cm^−2^. **Figure** **5**a shows the variation in CE during repeated galvanostatic Li plating/stripping tests. Although the CE of the Ni foil and bare Np‐3 electrodes rapidly dropped below 90% in the early stages of cycling (6th and 12th cycles for Ni foil and Np‐3, respectively), the cell employing the Ni_C_Ag anode stably operated for more than 60 cycles, which maintains a CE of over 97.9%. Moreover, as shown in Figure 5b, the Ni_C_Ag anode showed a nucleation overpotential close to zero (less than 1 mV), whereas the Ni foil and Np‐3 anode showed a considerably higher nucleation overpotential of 97.2 and 23.7 mV, respectively. We ascribed this reduced overpotential to the synergistically combined strategy of 3D architecture design and Ni particle modification with C and Ag.^[^ ^8^, ^21^ ^]^ The de‐alloying reaction signal around 0.4 V during stripping (Figure S11, Supporting Information) indicated that Ag formed Li‐Ag alloy during Li deposition. Though Ni_C_Ag showed notably improved stability and CE with reduced overpotential, the main cause of irreversibility in this system needs to be clarified. For this purpose, the Ni_C_Ag anode was examined after 10 cycles of Li deposition and striping. As shown in Figure S12, Supporting Information, there was no noticeable layer of side reaction products. On the other hand, the thickness of anode was increased to 20 µm which was 3 µm thicker than that of the anode after the first Li deposition and stripping (Figure 4h). Therefore, we consider that some of the Li deposits were trapped in Ni_C_Ag anode as dead Li and reduced CE. To clarify the interfacial properties depending on the Li deposition behavior, an electrochemical impedance spectroscopic (EIS) analysis for the cells with Ni foil, Np‐3, and Ni_C_Ag anodes in the pristine state before and after repeated Li deposition/stripping was performed. The impedance data were analyzed with an equivalent circuit shown in Figure S13, Supporting Information, and fitted resistance values are shown in the table. As shown in Figure 5c, the interfacial resistance of the cell with the Ni foil and Np‐3 increased remarkably after 5 cycles from 127.2 to 625.3 Ω cm^2^ for the Ni foil and from 36.5 to 1355.6 Ω cm^2^ for Np‐3. For both cells, the rapid increase in the interfacial resistance was attributed to the separation of the anode/LPS interface during Li deposition. However, Np‐3 anode showed a much faster increase in impedance compared to that of Ni foil. This might be due to the limited contact area between LPS SE and Np‐3 anode. Due to the rough surface of Np‐3 anode, the interfacial contact area between the Np‐3 anode and the SE was presumably smaller than that between the SE and the Ni foil with flat surface. The local current density at the interface between Np‐3 and SE could be thus higher than that at Ni foil and SE interface, and the higher local current density may induce much severe side reactions between Li deposits and the SE. Therefore, the cell with Np‐3 anode could show much rapid increase in interfacial resistance. The interfacial resistance of Ni_C_Ag at the pristine state was similar to that of Np‐3 (37.3 Ω cm^2^), and it only increased to 86.3 and 132.5 Ω cm^2^ after 5 and 10 cycles, respectively, which was more than 10 times smaller than that of Np‐3. The reduced interfacial resistance of the cell with the Ni_C_Ag anode originates from the complete storage of Li deposits within the Ni_C_Ag anode, and this results in a stable anode/LPS interface.

To verify the synergistic effect of optimized porous architecture and surface modification with carbon/silver, we fabricated Cu_C_Ag through the same procedure for Ni_C_Ag. The Cu particles with diameters around 200 nm were employed. As shown in Figure S14, Supporting Information, carbon layers evenly coated the surface of Cu particles, and Ag nanoparticles were homogeneously distributed. Unlike Ni_C_Ag with a graphitic carbon coating layer, the carbon layer of Cu_C_Ag appeared amorphous. We ascribed this difference to different catalytic effects between Ni and Cu for converting amorphous carbon into graphene.^[^ ^26^ ^]^ The Li deposition/stripping behavior of Cu_C_Ag was similar to that of Ni_C_Ag (Figure S15, Supporting Information). The Cu_C_Ag electrode can store the entire Li deposits of 2 mAh cm^−2^ within the anode without separation between the electrode and LPS SE. The thickness of Cu_C_Ag anode increased from 20 to 25 µm, and Li deposits filled the empty spaces after Li deposition (Figure S15a–e, Supporting Information). After Li stripping to 1 V, the thickness of anode decreased to 22 µm (Figure S15f, Supporting Information). During the repeated galvanostatic Li deposition/stripping tests in the half‐cell configuration, the cell with Cu_C_Ag electrode stably operated for 45 cycles with the curtailing capacity of 2 mAh cm^−2^ and at a 0.5 mA cm^−2^ current density (Figure S16, Supporting Information). Through the electrochemical tests of Cu_C_Ag, it was confirmed that the porous architecture with proper pore size and surface modification with carbon/silver was the pivotal factor that enables the stable Li deposition and stripping within anode without anode/SE separation.

**Figure 6:**
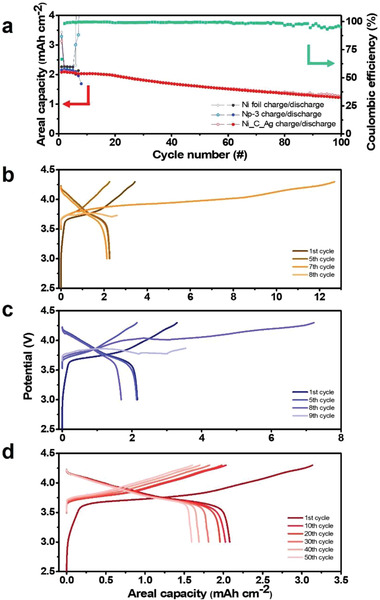
a) Cycling performance and CE of NCM full cell employing Ni foil, Ni nanoparticle, and Ni_C_Ag anode. b–d) Voltage profiles of the NCM full cell employing (b) Ni foil, (c) Ni nanoparticles (Np‐3), and (d) Ni_C_Ag anode.

Further improvement in electrochemical performance was achieved by employing an argyrodite‐type SE of Li_6_PS_5_Cl_0.5_Br_0.5_ (LPSClBr). The cell with LPSClBr was expected to show improved electrochemical performance and operate at a lower temperature of 30 °C because LPSClBr has a higher Li‐ion conductivity than LPS.^[^ ^27^, ^28^ ^]^ Figure S17, Supporting Information, shows SEM images after Li deposition and stripping within the cell employing the LPSClBr solid electrolyte. Consistent with the previous result of the cell employing LPS SE, all Li deposits were accommodated within the Ni_C_Ag anode, and the thickness of the anode increased from 14 to 21 µm (Figure S17a, Supporting Information). The corresponding EDS elemental mapping confirmed that the metallic Li layer did not separate the anode/LPSClBr interface (Figure S17e–g, Supporting Information). As shown in Figure S17b, Supporting Information, the anode and LPSClBr maintained their intimate contact after Li deposition. Individual anode particles were encased in Li deposits at both the SE and CC sides of the anode, and the Li deposits were observed in the top‐view SEM images of the CC side (Figure S17b–d, Supporting Information). The entire Ni_C_Ag anode participated in accommodating the Li deposits. After Li stripping to 1 V, the thickness of the anode decreased to 16 µm. Furthermore, when 3 mAh cm^−2^ Li was plated onto the Ni_C_Ag, the thickness of anode increased to 26 µm, and no Li deposits separated the interface between anode and argyrodite SE (Figure S18, Supporting Information). After further Li deposition to 4 mAh cm^−2^, Li deposits were observed at the interface of the anode/SE (Figure S19, Supporting Information). The thickness of the plated Li at the interface was around 3 µm, thus the maximum areal capacity of Ni_C_Ag anode is expected around 3.4 mAh cm^−2^. The CEs during repeated galvanostatic Li plating/stripping tests in the half‐cell configuration are plotted in Figure S20a, Supporting Information; the voltage profiles at 1st Li deposition are shown in Figure S20b, Supporting Information. Owing to its higher Li ion conductivity, deposition overpotentials significantly decreased compared to those of the cells employing LPS SE (10–15 mV in Figure S13b, Supporting Information, vs 100–150 mV in Figure 5b). As in the cells with LPS SE, the Ni_C_Ag anode presented a significantly lower nucleation overpotential compared to those of the Ni foil and Np‐3 anodes. Furthermore, cells with Ni_C_Ag cycled more than 100 times maintaining a CE of 96.9%. The electrochemical performance of the Ni_C_Ag anode was investigated in a Li‐free full cell by employing LiNbO_3_‐coated LiNi_0.8_Co_0.1_Mn_0.1_O_2_ (NCM811) as the cathode. The mass loading of the cathode active material was 17 mg cm^−2^, and the cells were cycled at a current density of 40 mA g_cathode_^−1^ (0.68 mA cm^−2^) for both charge and discharge between 3 and 4.3 V. As shown in **Figure** **6**b,c, the short‐circuit occurred in both cells with Ni foil and Np‐3 anodes at the 8th cycle. We ascribed this early short circuit to the unstable interface between the anode and SE caused by the separation of the interface. Subsequent contact loss during Li deposition and stripping may lead to inhomogeneous Li deposition and high local current density, which results in dendritic Li growth and short‐circuit.^[^ ^29^ ^]^ In contrast, the full cell with the Ni_C_Ag anode showed a considerably more stable cycling performance (Figure 6a,d). The full cell with the Ni_C_Ag anode cycled stably with a capacity retention of 58.83% after 100 cycles with an average CE of 99.47%. The full cell delivered an areal discharge capacity of 2.08 mAh cm^−2^ at the initial discharge, and 1.22 mAh cm^−2^ at the 100th cycle. The greatly improved cycle stability and CE were attributed to the stable anode/SE interface and reduced nucleation overpotential.

## 3 Conclusions

We investigated the Li deposition behavior depending on the pore size of the 3D anode and designed a highly reversible 3D porous anode for Li‐free all‐solid‐state batteries. The Li deposits were plated within the 3D Ni particle anode more easily in the anode with a smaller pore size expanding the volume of the anode, whereas a larger amount of Li was deposited at the anode/SE interface when the pore size was large. By comparing the size‐dependent Li deposition behavior, the advance of metallic Li to the 3D porous anode under the given operating temperature and pressure was considered via diffusional Coble creep. To further improve Li storage capacity in Ni‐based 3D anode, we modified the Ni particles by carbon coating and Ag nanoparticle decoration to facilitate Li movement and reduce the energy barrier for Li nucleation. The resulting Ni_C_Ag 3D anode successfully accommodated the entire Li deposit of 2 mAh cm^−2^ within the porous architecture without the separation of the anode/SE interface. With the significantly reduced nucleation overpotential and interfacial resistance, the Ni_C_Ag anode showed the high reversibility of Li deposition and stripping. The Ni_C_Ag anode could be cycled for more than 60 and 100 cycles with LPS and LPSClBr SE in half cells with a capacity limit of 2 mAh cm^−2^ and a current density of 0.5 mA cm^−2^ maintaining a CE of 97.9% and 96.9%, respectively. Further, the synergistic effects of the stable anode/SE interface and reduced nucleation energy barrier enabled stable NCM full‐cell cycling at a room temperature of 30 °C. The NCM811 cathode/Ni_C_Ag anode full cell in the Li‐free configuration showed an initial areal discharge capacity of 2 mAh cm^−2^ and operated stably with a CE of 99.47% for 100 cycles.

This work underpinned our fundamental understanding of Li movement in porous anodes in solid‐state batteries and demonstrated that maintaining the intimate contact between the anode and SE by employing a suitable 3D architecture is crucial for the stable operation of Li‐free solid‐state batteries.

## 4 Experimental Section

### Synthesis of Ni_C_Ag

After 1 g of Ni powder (Np‐3, Sigma‐Aldrich) was dispersed in 100 mL of triethylene glycol (Sigma‐Aldrich), the mixture was stirred at 220 °C for 14 h to coat the polymer layer on the Ni particles. The resulting suspension was washed with ethanol five times by centrifugation and dried overnight at 80 °C in a vacuum oven. For Ag nanoparticle decoration, 500 mg of the polymer‐coated Ni powder was dispersed in 140 mL of deionized (DI) water, and 100 mg of AgNO_3_ (Sigma‐Aldrich) was added to the suspension. After stirring for 10 min, 50 mL of a 1 m KOH solution was slowly dropped into the suspension, and 50 mL of 1 m ethylene glycol (Sigma‐Aldrich) was added. Then, the suspension was stirred at 80 °C for 3 h and washed with DI water five times by centrifugation. The resulting powder was dried overnight at 80 °C in a vacuum oven. To carbonize the polymer layer, dried powder was annealed in a tube furnace with the following temperature profile under Ar atmosphere: heat to 350 °C at a ramping rate of 2 °C min^−1^, maintain temperature at 350 °C for 1 h, heat to 700 °C at a ramping rate of 2 °C min^−1^, and maintained at 700 °C for 4 h. The resulting Ni_C_Ag powder was stored in a vacuum oven at 80 °C until use.

### Cell Fabrication

Ni particle‐based electrodes were fabricated by casting a slurry composed of Ni particles (Sigma‐Aldrich) or Ni_C_Ag and polyvinylidene fluoride (PVDF, Sigma‐Aldrich) in NMP (DAEJUNG Chemicals) with a weight ratio of 99:1. The slurry was cast on a Ni foil CC with a thickness of 10 µm and dried in an oven at 120 °C overnight. The loading of Ni_C_Ag was 8.9 mg cm^−2^. A total of 90 mg of Li_3_PS_4_ (LPS, NEI corporation) or Li_6_PS_5_Cl_0.5_Br_0.5_ (LPSClBr, supplied by Hyundai Motor Company) was pressed into pellets in a polyether ether ketone mold with an inner diameter of 10 mm under an uniaxial pressure of 380 MPa at room temperature to assemble ASSBs. The cast electrodes were assembled on the prepared SE pellet and repressed at 200 MPa. For the half‐cell, the Li foils with a thickness of 200 µm were assembled on the other side of the pellet and pressed to an operating pressure of 5 MPa. For full cell preparation, the cathode mixture was assembled on the other side of the LPSClBr SE pellet and pressed under a uniaxial pressure of 380 MPa. The NCM cathode mixture was composed of LiNbO_3_‐coated LiNi_0.8_Co_0.1_Mn_0.1_O_2_ (1 wt.%) (Ampcera), LPSClBr, and Super C65 carbon (TIMCAL) with a mass ratio of 70:30:3. The mass loading of the cathode active material was 17 mg cm^−2^. For full cell test, the anodes were precycled from 0 to 1 V for 10 cycles and the potential was dropped to 0 V with a current density of 0.5 mA cm^−2^ in the half‐cell with liquid electrolyte before being assembled in ASSBs to activate the anode. The cell with the liquid electrolyte used two layers of commercial separators (Celgard 2400) and liquid electrolytes of 1 m lithium bis(trifluoromethylsulfonyl)imide (LiTFSI, Sigma‐Aldrich) in 1,3‐dioxolane/1,2‐dimethoxyethane (DOL/DME, 1/1:v/v, Sigma‐Aldrich) with 1% LiNO_3_ (Sigma‐Aldrich). After cycling, the cell was disassembled and the anode was washed with DOL and dried under vacuum. Then, the anode was assembled onto the other side of the ASSB and repressed at 200 MPa. The resulting NCM full cell was subjected to uniaxial pressure using a custom‐made screw with a stainless‐steel framework of 490 N cm (≈30 MPa).

### Characterization

The morphology, composition of materials, and Li‐deposited ASSBs were observed using SEM (Nova Nano SEM 450) with an EDS detector. The morphology and crystal structure were further characterized by high‐resolution TEM (JEM 2100F, JEOL) with an EDS detector. The pore size distribution of the Ni particle electrodes was analyzed using mercury porosimetry (Autopore V 9600). The electrochemical impedance was measured using EIS (BioLogic, SP‐200) in the frequency range of 7 MHz to 100 mHz.

## Conflict of Interest

The authors declare no conflict of interest.

## Supporting information

Supporting Information

## Data Availability

The data that support the findings of this study are available from the corresponding author upon reasonable request.
