# Supplementary material for: Designing 3D Anode Based on Pore‐Size‐Dependent Li Deposition Behavior for Reversible Li‐Free All‐Solid‐State Batteries
Source: Adv Sci (Weinh). 2022 Aug 10;9(28):2203130. doi: 10.1002/advs.202203130 (PMC9534956; doi:10.1002/advs.202203130)
Supplement: Supplementary file 1 — Supporting Information [file ADVS-9-2203130-s001.pdf]

ADVANCED SCIENCE
Open Access

Supporting Information

for *Adv. Sci.*, DOI 10.1002/advs.202203130

Designing 3D Anode Based on Pore-Size-Dependent Li Deposition Behavior for Reversible Li-Free All-Solid-State Batteries

*Se Hwan Park, Dayoung Jun, Gyu Hyeon Lee, Seong Gyu Lee, Ji Eun Jung, Ki Yoon Bae, Samick Son and Yun Jung Lee**

Supporting Information

Designing 3D Anode Based on Pore-Size-Dependent Li Deposition Behavior for Reversible Li-Free All-Solid-State Batteries

*Se Hwan Park, Dayoung Jun, Gyu Hyeon Lee, Seong Gyu Lee, Ji Eun Jung, Ki Yoon Bae, Samick Son, and Yun Jung Lee**

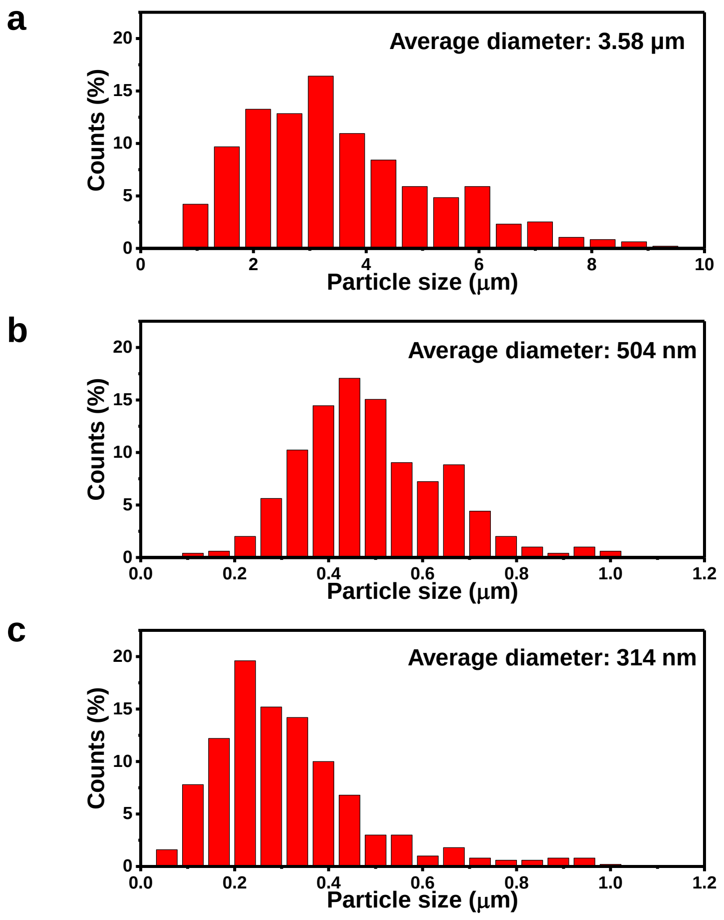

Figure S1. Size distribution of Ni particles: (a) Np-1, (b) Np-2, and (c) Np-3.

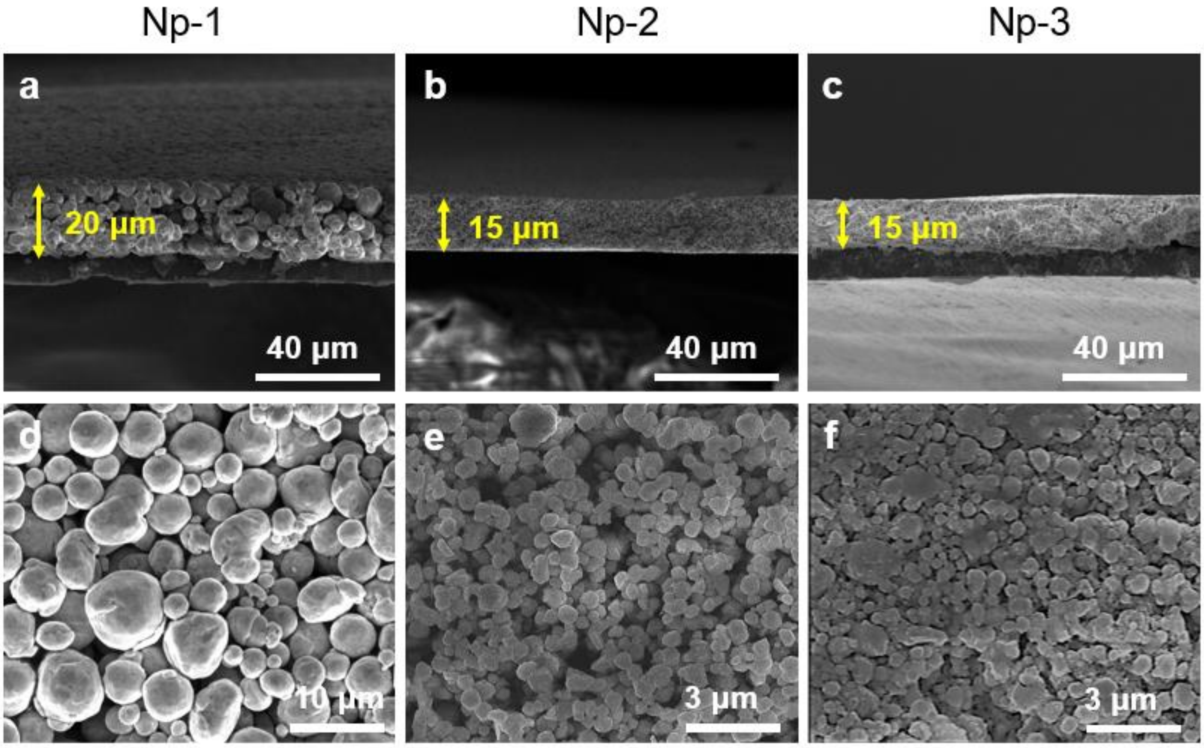

Figure S2. Cross section (a-c) and surface (d-f) SEM images of 3D Ni electrodes composed of different Ni particles. (a and d) Np-1, (b and e) Np-2, and (c and f) Np-3.

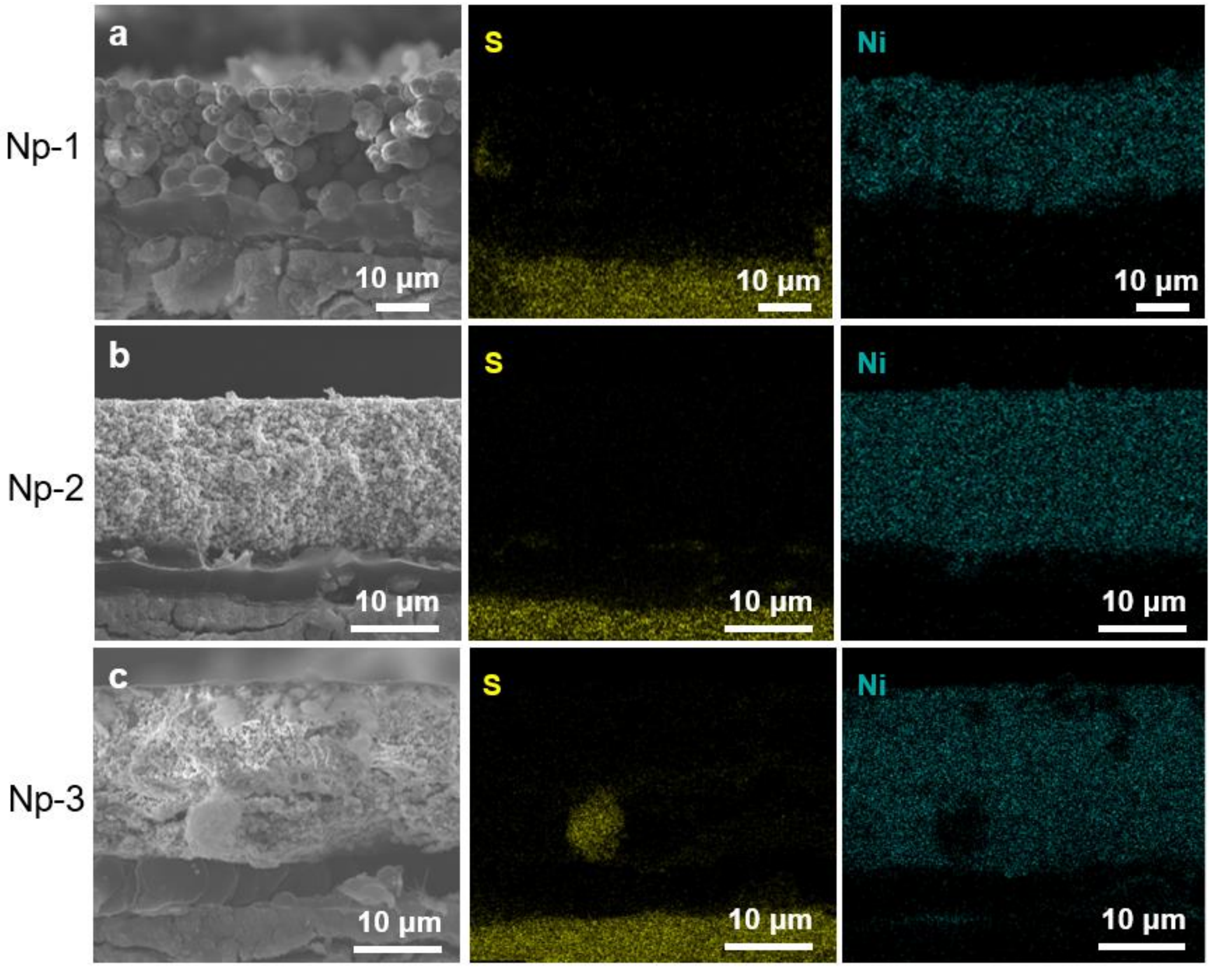

Figure S3. SEM images and EDS elemental mappings for Li deposited 3D Ni electrodes plated to the capacity of 2 mAh cm^{-2}. (a) Np-1, (b) Np-2, and (c) Np-3.

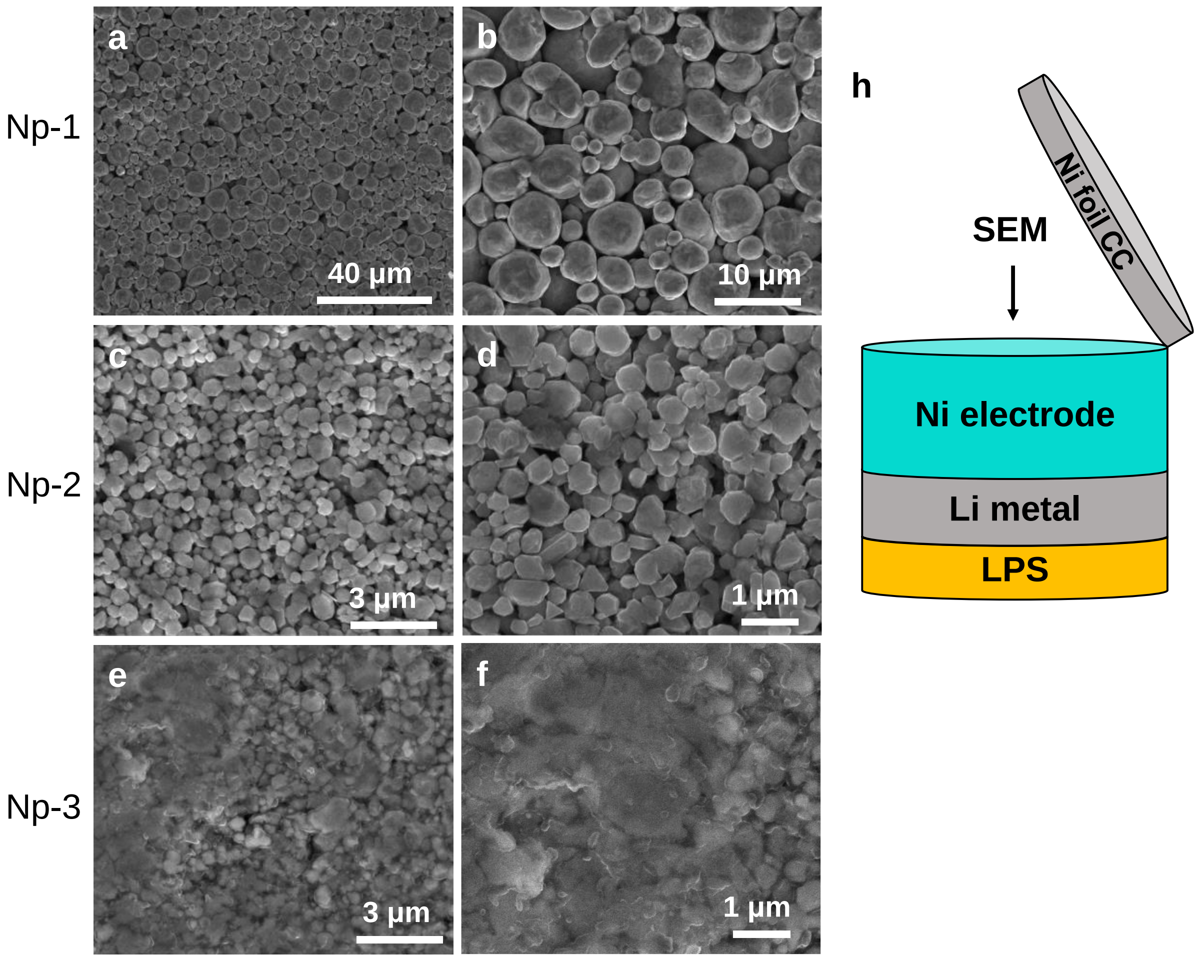

Figure S4. (a-f) SEM images of Li deposited 3D Ni electrodes plated to the capacity of 2 mAh cm^{-2} observed at the current collector side after delaminating the current collector as shown in schematic illustration of (h). (a and b) Np-1, (c and d) Np-2, and (e and f) Np-3.

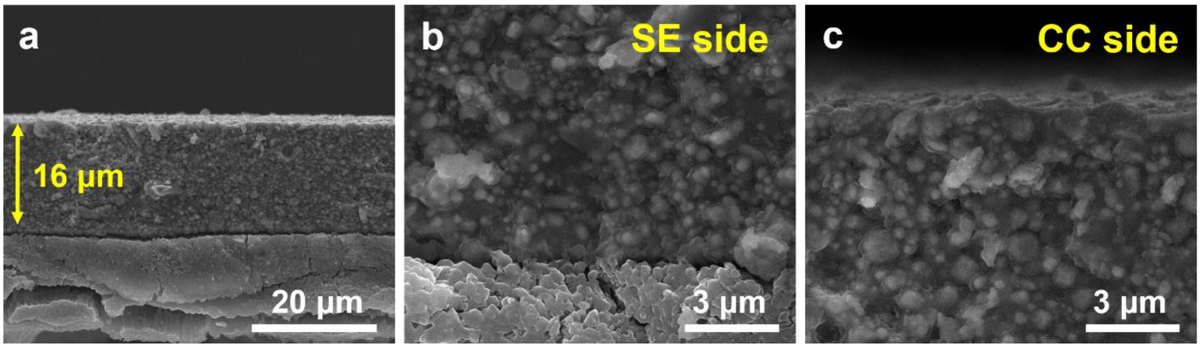

Figure S5. SEM images of Li deposited Np-3 electrodes plated to the capacity of 0.5 mAh cm^{-2}. (b) and (c) represent the Ni electrode of solid electrolyte and current collector side, respectively.

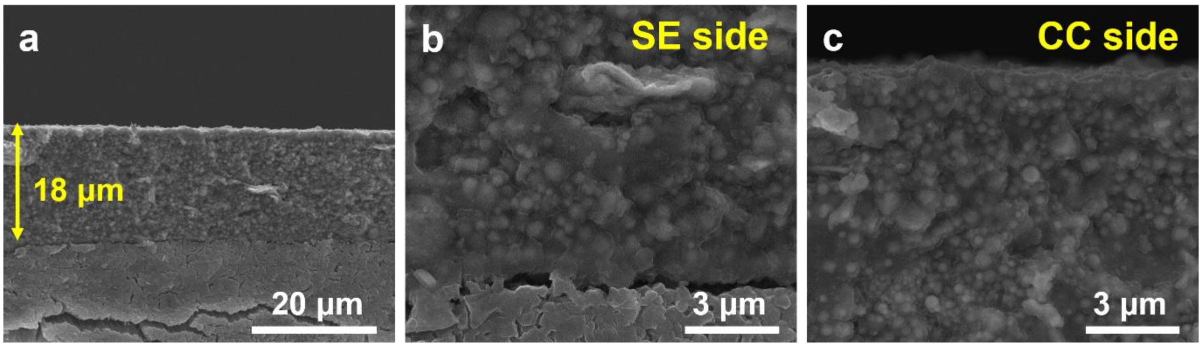

Figure S6. SEM images of Li deposited Np-3 electrodes plated to the capacity of 1.0 mAh cm^{-2}. (b) and (c) represent the Ni electrode of solid electrolyte and current collector side, respectively.

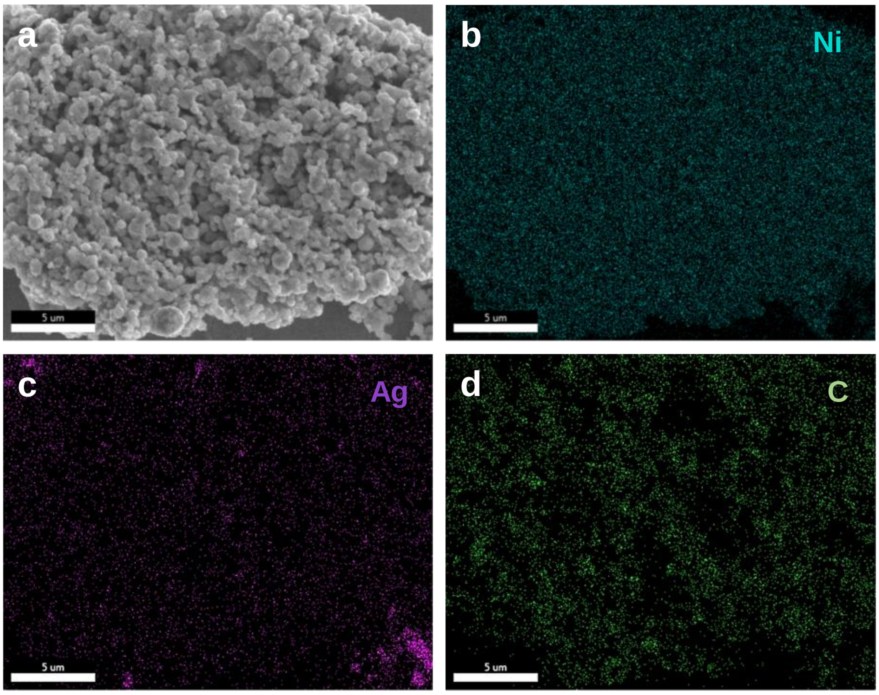

Figure S7. (a) SEM image of Ni_C_Ag powder and corresponding EDS elemental mappings for (b) Ni, (c) Ag, and (d) Ni.

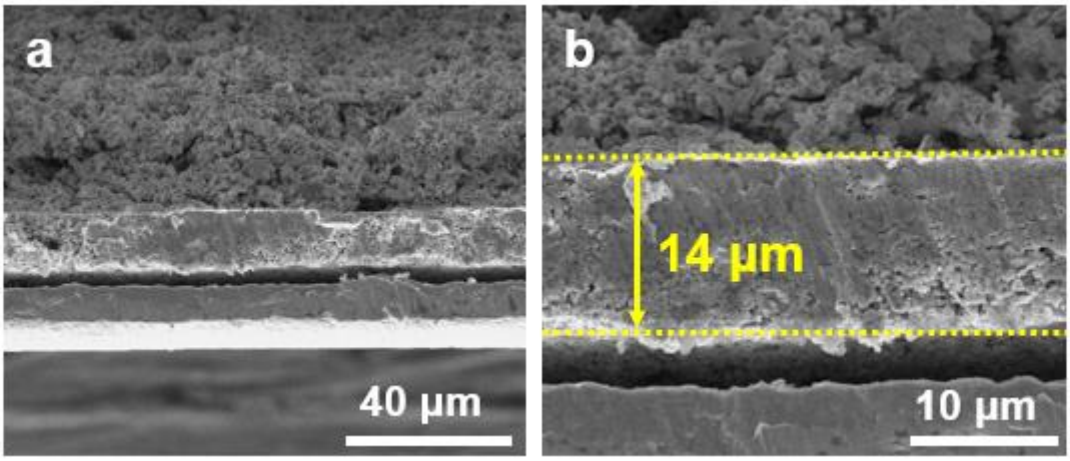

Figure S8. Cross sectional SEM image of the Ni_C_Ag electrode

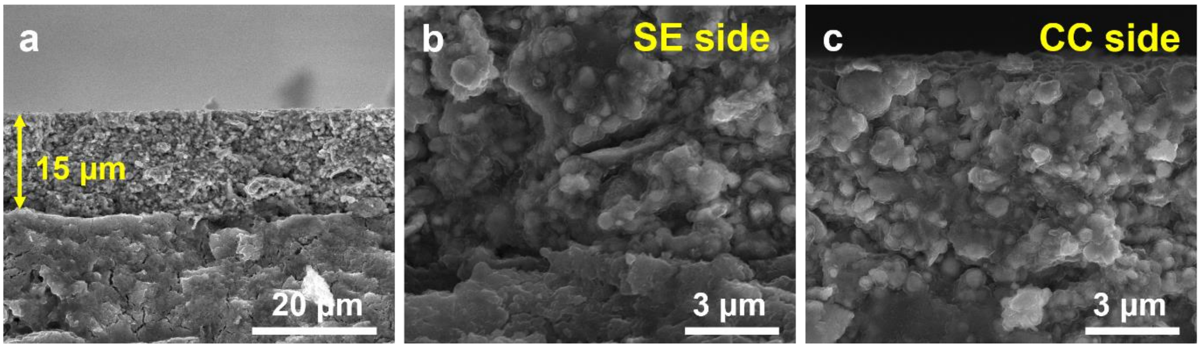

Figure S9. SEM images of Li deposited Ni_C_Ag electrodes plated to the capacity of 0.5 mAh cm^{-2}. (b) and (c) represent the Ni electrode of solid electrolyte and current collector side, respectively.

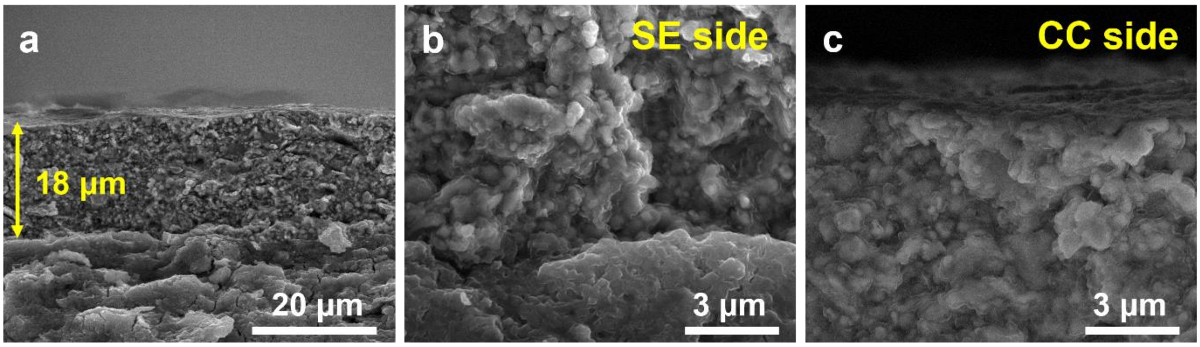

Figure S10. SEM images of Li deposited Ni_C_Ag electrodes plated to the capacity of 1.0 mAh cm^{-2}. (b) and (c) represent the Ni electrode of solid electrolyte and current collector side, respectively.

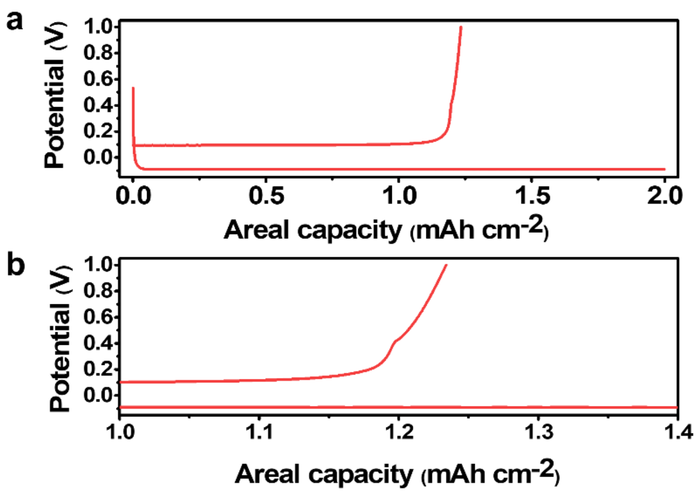

Figure S11. (a) The 1st Li deposition and stripping voltage profiles of Ni_C_Ag electrode. (b) Magnified voltage profile of Figure S11a.

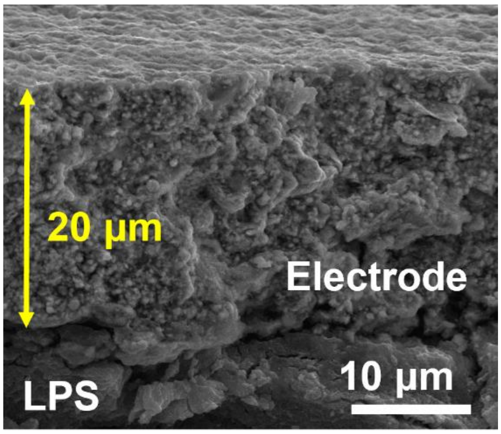

Figure S12. Cross sectional SEM image of Ni_C_Ag electrode after 10 cycles of 2 mAh cm^{-2} Li plating and stripping to 1 V.

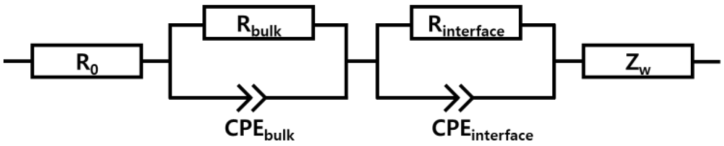

	Resistance (Ω cm^2)	Ni foil	Np-3	Ni_C_Ag
Pristine	R_{bulk}	276.7	248.2	277.4
	$R_{interface}$	127.2	36.5	37.3
After 5 cycles	R_{bulk}	519.6	343.4	337.9
	$R_{interface}$	625.3	1355.6	86.3
After 10 cycles	R_{bulk}			401.2
	$R_{interface}$			132.5

Figure S13. Equivalent circuit employed to analyze impedance in Figure 5C and fitted resistance value.

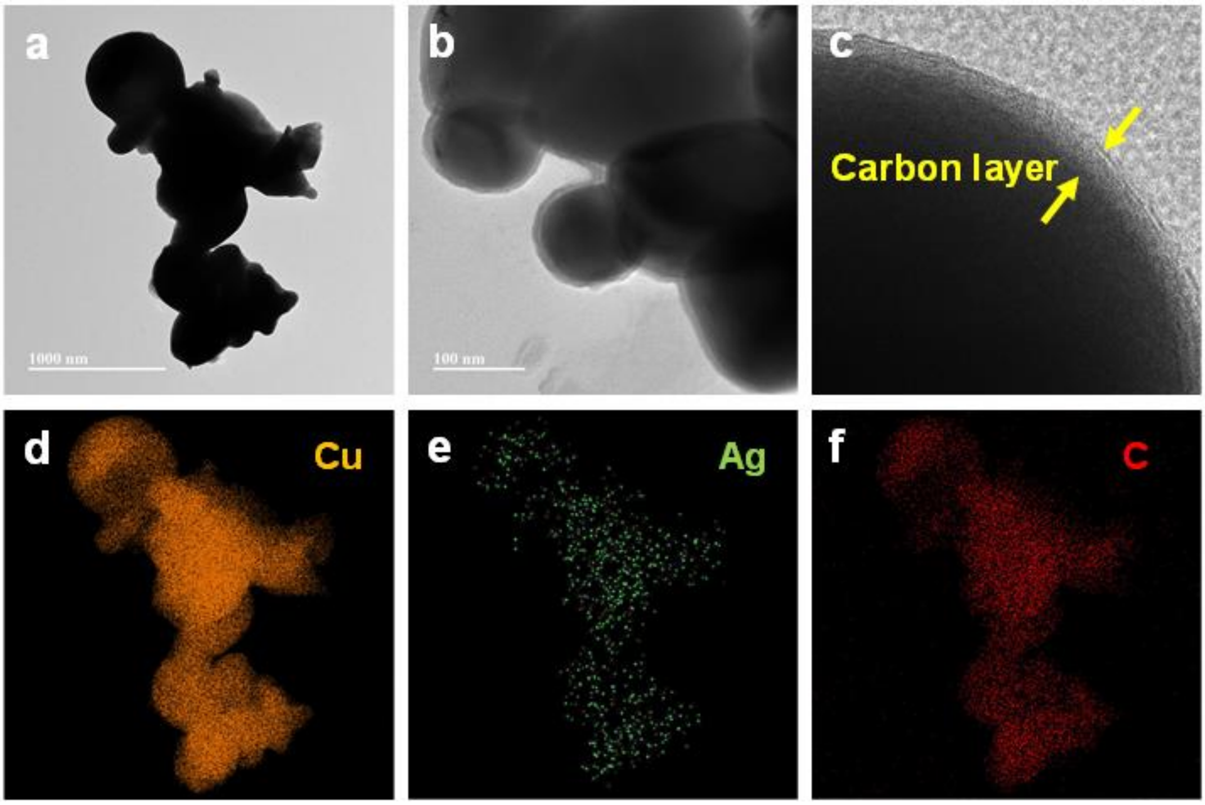

Figure S14. (a-c) TEM images of Cu_C_Ag powder and corresponding EDS elemental mappings for (d) Cu, (e) Ag, and (f) C

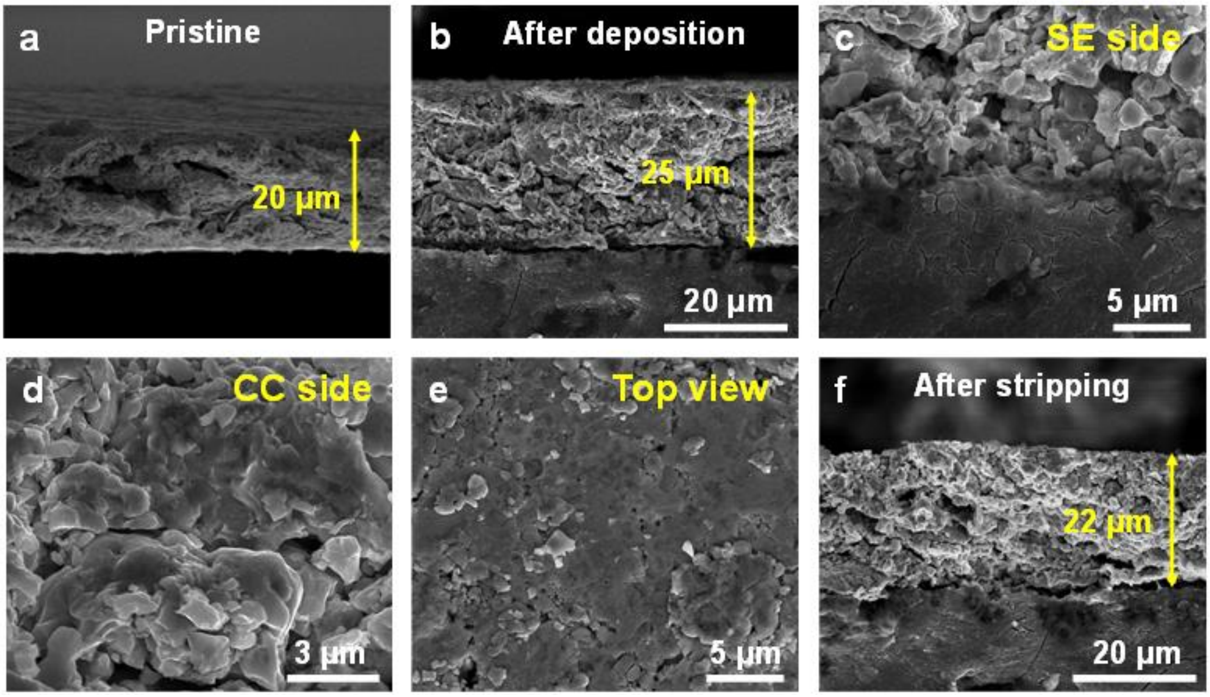

Figure S15. (a) Cross sectional SEM image of the Cu_C_Ag electrode. (b-d) Cross section and (e) surface SEM image of Li deposited Cu_C_Ag electrode plated to the capacity of 2 mAh cm^{-2}: (c) for solid electrolyte side, (d) for current collector side, (e) for surface of current collector side observed after delaminating Ni foil current collector. (f) Cross sectional SEM image of Cu_C_Ag electrode after 2 mAh cm^{-2} Li plating and stripping to 1 V.

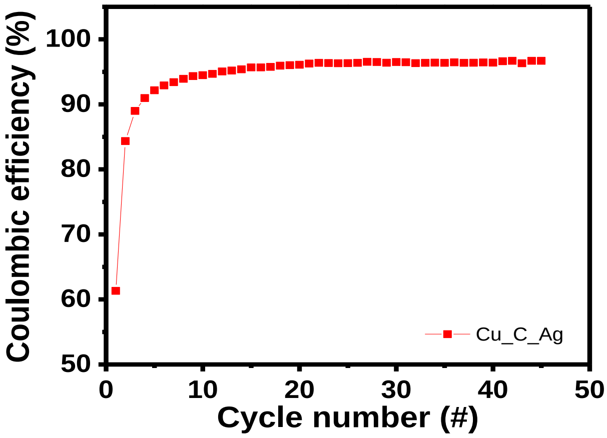

Figure S16. Variation of coulombic efficiency during the repeated Li plating and stripping Cu_C_Ag electrode with a curtailing capacity of 2 mAh cm^{-2} and stripped to 1 V at a current density of 0.5 mA cm^{-2}.

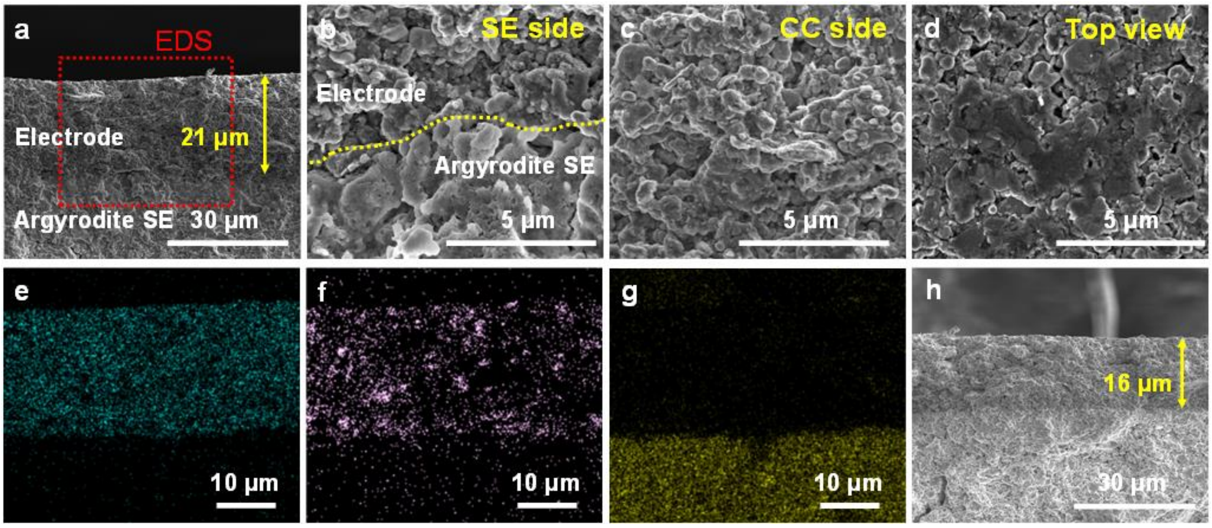

Figure S17. (a-c) Cross section and (d) top-view SEM images and (e-g) correspond EDS elemental mappings of Li deposited Ni_C_Ag electrode plated to the capacity of 2 mAh cm^{-2} within solid-state battery employing argyrodite type electrolyte: (b) for solid electrolyte side, (c) for current collector side, (d) for top-view of current collector side observed after delaminating Ni foil current collector (refer to the schematic illustration in Figure S4h). EDS elemental mappings for (e) Ni, (f) Ag, and (g) S. (h) Cross section SEM image of Ni_C_Ag electrode after 2 mAh cm^{-2} Li plating and stripping to 1 V.

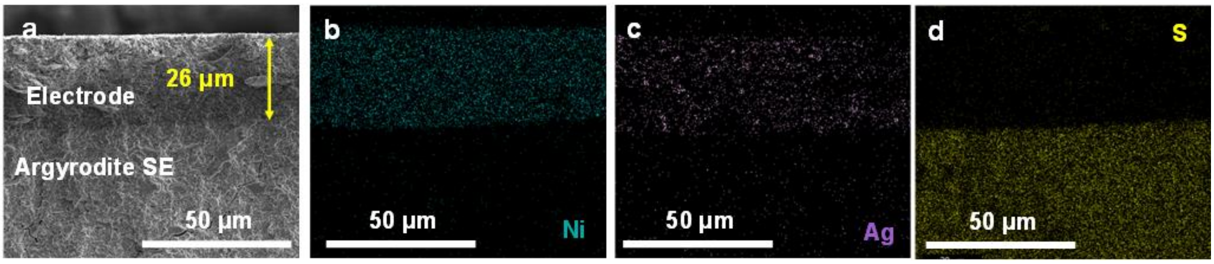

Figure S18. (a) Cross section and (b-d) corresponding EDS elemental mappings of Li deposited Ni_C_Ag electrode plated to the capacity of 3 mAh cm^{-2} within solid-state battery employing argyrodite type electrolyte. EDS elemental mappings for (b) Ni, (c) Ag, and (d) S.

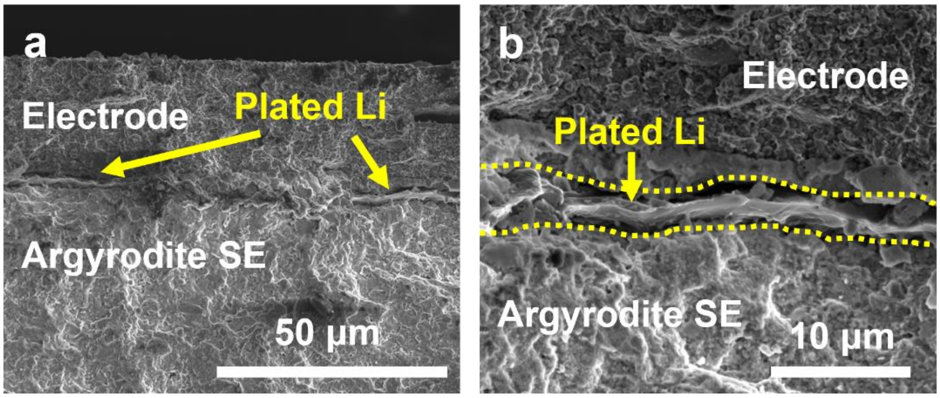

Figure S19. Cross sectional SEM images of Li deposited Ni_C_Ag electrode plated to the capacity of 4 mAh cm^{-2} within solid-state battery employing argyrodite type electrolyte.

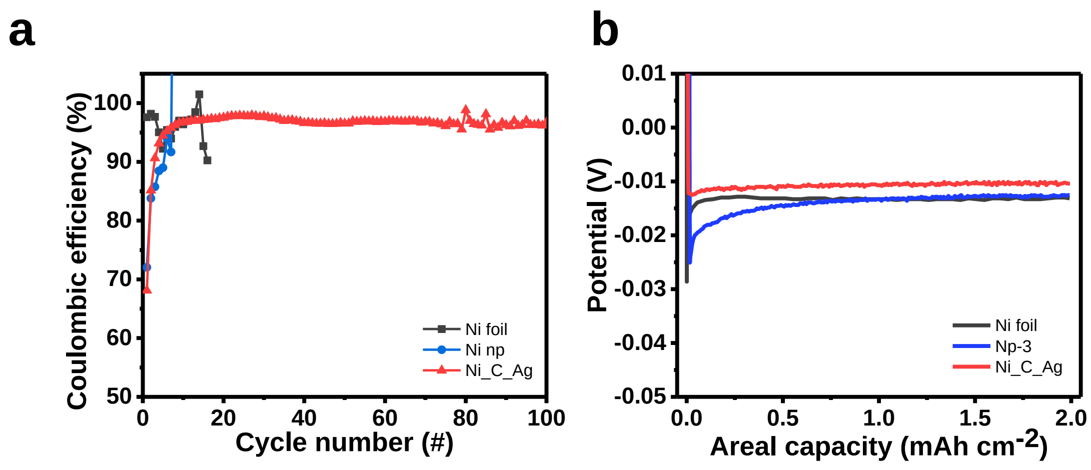

Figure S20. (a) Variation of Coulombic efficiency during the repeated Li plating and stripping on Ni foil, Np-3, and Ni_C_Ag electrode within solid-state battery employing argyrodite type electrolyte. (b) 1st Li deposition voltage profiles of Ni foil, Np-3, and Ni_C_Ag electrode.
